# HIF-2α Controls Expression and Intracellular Trafficking of the α2-Subunit of Na,K-ATPase in Hypoxic H9c2 Cardiomyocytes

**DOI:** 10.3390/biomedicines11112879

**Published:** 2023-10-24

**Authors:** Emel Baloglu

**Affiliations:** Department of Medical Pharmacology, School of Medicine, Acibadem Mehmet Ali Aydinlar University, 34752 Istanbul, Turkey; emel.baloglu@acibadem.edu.tr; Tel.: +90-216-500-4048

**Keywords:** heart failure, ischemic heart disease, hypoxia, Na,K-ATPase, HIF, cardiac glycosides, H9c2 cells

## Abstract

The Na,K-ATPase (NKA) pump plays essential roles for optimal function of the heart. NKA activity decreases in necropsy materials from ischemic heart disease, heart failure and in experimental models. Cellular adaptation to hypoxia is regulated by hypoxia-induced transcription factors (HIF); we tested whether HIFs are involved in regulating the expression and intracellular dynamics of the α2-isoform of NKA (α2-NKA). HIF-1α and HIF-2α expression was suppressed in H9c2 cardiomyocytes by adenoviral infection, where cells were kept in 1% O_2_ for 24 h. The silencing efficiency of HIFs was tested on the mRNA and protein expression. We measured the mRNA expression of α2-NKA in HIF-silenced and hypoxia-exposed cells. The membrane and intracellular expression of α2-NKA was measured after labelling the cell surface with NHS-SS-biotin, immunoprecipitation and Western blotting. Hypoxia increased the mRNA expression of α2-NKA 5-fold compared to normoxic cells in an HIF-2α-sensitive manner. The plasma membrane expression of α2-NKA increased in hypoxia by 2-fold and was fully prevented by HIF-2α silencing. Intracellular expression of α2-NKA was not affected. These results showed for the first time that in hypoxic cardiomyocytes α2-NKA is transcriptionally and translationally regulated by HIF-2α. The molecular mechanism behind this regulation needs further investigation.

## 1. Introduction

The Na,K-ATPase (NKA) pump is a member of the P-type ATPase family [1]. In the heart muscle it maintains the generation of action potentials, electrical activity, maintenance of Na^+^ and Ca^2+^ ion gradients, and cell survival [2]. Thus, proper function of the NKA is vital for cellular viability. NKA is also the target of cardiac glycosides, used as anti-arrhythmic and positive inotropic drugs for some patients with end-stage heart failure.

The well-known function of the NKA is to transport 3Na^+^ ions out of the cell and 2K^+^ ions into the cell by the energy obtained from ATP hydrolysis. NKA in structure consists of a catalytic α subunit, a regulatory β subunit controlling membrane-insertion of α-NKA, and a γ subunit, which is a member of the FXYD proteins regulating the function of NKA [3].

The α-NKA has binding sites for Na^+^, K^+^, Mg^2+^, ATP and cardiac glycosides [4]. Its four isoforms (i.e., α1, α2, α3 and α4) are expressed in different abundances in different tissues and species [5,6]. Among them, α1-NKA is the ubiquitous form, and it is expressed in all cell types; the α2 and α3 isoforms are mostly expressed in the heart, skeletal muscle, and neurons. In human ventricular myocardium, α1, α2 and α3 isoforms are predominantly expressed; in mouse and rat myocardium, only α1- and α2-NKA are expressed [6]. The β subunit has three isoforms (i.e., β1, β2 and β3). The most studied β1isoform controls the plasma membrane insertion of α-NKA [5]. Thus β1-NKA is required for the functionality of NKA.

In heart ventricular myocytes, α1-NKA is densely and diffusely located in the plasma membrane, whereas α2 and α3 subunits are mostly found in T-tubules [7]. The characteristic expression patterns of the NKA isoforms suggest the relevance of the NKA activity in physiological and pathological conditions.

The expression of α2-NKA is strictly regulated during development in a tissue-specific way [8]. It has been considered as the minor isoform of the NKA expressed in the cardiovascular system. Although α1-NKA appears to be the major and house-keeping form of NKA, recent studies have identified that α2-NKA plays critical roles in the regulation of systolic blood pressure [9] and myocardial response to pressure overload [10,11,12]. However, less is known about its regulation and its role in the activity of NKA in ischemic heart diseases.

Studies in necropsy materials from patients with heart failure of different aetiology reported decreased activity of NKA [13,14], which resulted in the elevation of intracellular Na^+^ and Ca^2+^ levels, deterioration of diastolic function, and arrhythmia [15,16]. Similar results have been shown in various animal models of ischemic heart disease as well [13,17,18,19]. The decreased activity of the NKA has been associated with altered mRNA and protein expression of the α1, α2 and β1 isoforms [13,14,18,20]. However, information about the regulation of α2-NKA in the ischemic myocardium is scarce and discrepant. For instance, in the ventricles of rabbits which developed heart failure and the myocardial infarction in rats, α2-NKA protein expression decreased [18,20]. In the right atrium of patients with hypertension, α2-NKA mRNA expression increased 5-fold [21], whereas in the myocardium of patients with ischemic or dilated cardiomyopathy, there was no change in the mRNA or protein expression [22]. In contrast, in a renovascular hypertension model, α2-NKA mRNA and protein levels decreased [23] Zahler et.al. compared the mRNA expressions of α1, α2 and α3 isoforms in human ventricular tissue from normal and human diseased hearts and reported increased expression of the α3 isoform in failing human hearts, but a decreased expression of the three isoforms in pressure-overloaded right ventricles [24].

Ischemic heart disease results in hypoxia of the peripheral tissues, but, importantly, also of the myocardium. Hypoxia-induced transcription factors (HIFs) play a central role in the adaptation to hypoxia [25,26]. Increased levels of HIFs are common in myocardial ventricular biopsy specimens from patients undergoing coronary bypass surgery [27]; in human skin from hypertensive patients [28]; and in animal models of myocardial infarction [29,30], cardiac hypertrophy [31,32], arrhythmia [30], and pulmonary hypertension [33].

The aim of this study is to investigate whether the expression of α2-NKA is affected by hypoxia and HIFs using in-vitro culture of rat ventricular H9c2 cells as a model. Here, we report for the first time that hypoxia increases the mRNA and membrane expression of α2-NKA in HIF-2α-dependent manner.

## 2. Materials and Methods

### 2.1. Cell Culture

H9c2 cells (rat ventricular cardiomyocytes, ATCC, Manassas, VA, USA) were cultured in complete DMEM (c-DMEM) (Thermo Scientific, Waltham, MA, USA, #31885023) containing 1 g/L glucose, 4 mM L-glutamine, 1 mM Na-pyruvate, supplemented with 100 IU penicillin–streptomycin (Sigma, St. Louis, MO, USA, #P4433), 5 mM HEPES (#HEP.B Capricorn, Ebsdorfergrund, Germany) and 10% foetal bovine serum (# FBS-11A, Capricorn, Ebsdorfergrund, Germany) in an incubator with ambient air at 5% CO_2_ at 37 °C (normoxia). The medium was replaced every two days. The cells were grown until confluency reached 80%. For the experiments, cells were seeded at a density of 2 × 10^5^/cm^2^ in tissue culture plates and infected with adenoviral vectors 24 h after plating.

### 2.2. HIF Silencing Experiments and the In Vitro Ischemic Heart Model

Adenoviral vectors containing shRNA sequences to silence rat HIF-1α, HIF-2α and a scrambled sequence (scr.co) serving as control were generated as previously reported [34]. H9c2 cells were infected with 100 MOI (multiplicity of infection) in c-DMEM in the absence of antibiotics for 16 h, and then the medium was replaced with c-DMEM. Cells were then exposed to hypoxia in a modular chamber (MIC-101, Billups-Rothenberg) in 1% O_2_ + 5% CO_2_, residual gas N_2_ at 37 °C, while normoxic control cells were kept in 19% O_2_. The silencing efficiency of the HIFs was tested at the mRNA and protein levels in nuclear extracts after 2 h, 4 h, 6 h, and 24 h of hypoxia exposure. All other experiments were performed on cells exposed to 1% O_2_ for 24 h.

### 2.3. RNA Isolation and Quantitative RT-PCR

H9c2 cells were washed with phosphate-buffered saline (PBS) and lysed using the RLT reagent (Qiagen, Hilden, Germany). Total RNA was isolated with the RNeasy micro kit that included a DNase digestion step (Qiagen, Hilden, Germany) according to the manufacturer’s instructions. RNA was transcribed with the SensiFAST cDNA Synthesis Kit (#BIO-65054, Bioline, London, UK). Real-time quantitative PCR was performed in the CFX96 Touch Real-Time PCR Detection System (Biorad, Hercules, CA, USA) using the SensiFAST SYBR No-ROX (Bioline, #BIO-98005) and QuantiTect^®^ primers according to manufacturer’s instructions (QuantiTect^®^, Qiagen, Hilden, Germany). Results were analysed using the delta-delta CT method [35] with 28SrRNA as a reference gene representing normalized values for the normoxic scrambled control cells. The list of primers used is given in Table 1 (the sequences are not available due to company policy).

### 2.4. Preparation of Nuclear Extracts

After exposure to hypoxia for the indicated time periods, cells were washed with ice-cold PBS, lysed with a buffer composed of (in mM) 150 NaCl, 50 Tris (pH: 7.5), 5 EDTA, 0.5% NP-40, 1% Triton-X100, 1 DTT, 10 NaF, 1 NaVO_3_, and 1× protease inhibitor cocktail (Roche, #11836170001, Mannheim, Germany) and centrifuged at 12,000× *g* for 1 min at 4 °C. Nuclei were lysed with a buffer composed of (in mM) 300 NaCl, 50 KCl, 50 Hepes-KOH (pH: 7.9), 0.1 EDTA, 10% Glycerol, 1 DTT, 10 NaF, 1 NaVO_3_, and 1× protease inhibitor cocktail (Roche, Tokyo, Japan, #11836170001) by incubation for 30 min at 4 °C and vortexed repeatedly. Lysates were collected after centrifugation at 12,000× *g* for 20 min at 4 °C, aliquoted, and immediately frozen at −80 °C. Total protein was measured using the Bradford reagent (Bio-Rad Protein Assay Kit II, #1610731).

### 2.5. Cell Surface Biotinylation Experiments

The cell surface expression of α2-NKA was measured after biotinylation of membrane proteins by cell-impermeable EZ-Link Sulfo-NHS-SS-Biotin (Thermo Scientific, #21331), followed by immunoprecipitation and Western blotting. Cells were washed three times with ice-cold PBS, incubated with 1 μg/mL of Sulfo-NHS-SS-Biotin for 20 min at 4 °C, and washed three times with PBS containing glycine (100 mM). Cells were lysed with a buffer composed of 1% Triton X-100, 150 mM NaCl, 5 mM EDTA, 50 mM Tris, pH 7.5, and 1× protease inhibitor cocktail and centrifuged at 14,000× *g* for 20 min at 4 °C. Proteins were measured and supernatants containing 150–200 µg of total protein were incubated with 100 μL of 50% slurry of streptavidin-agarose beads (Pierce™ Streptavidin Agarose, Dallas, TX, USA, #20349) overnight at 4 °C. Beads were pelleted by centrifugation at 12,000× *g* for 10 min at 4 °C. Supernatants containing the non-biotinylated fraction representing the intracellular proteins were collected. The beads were washed three times with lysis buffer, and biotinylated proteins representing the membrane fraction were eluted by heating to 95 °C for 5 min in 2× Laemmli sample buffer. Equal amounts of non-biotinylated and biotinylated samples were used for SDS-PAGE and Western blotting. The success of cell surface biotinylation was evaluated by only detecting beta-actin in the intracellular pool and not the surface (biotinylated) membrane fraction.

### 2.6. Western Blotting

To measure HIF-1α and HIF-2α expression, 10–25 μg of nuclear extracts were separated on 10% SDS-PAGE and transferred onto nitrocellulose or PVDF membranes for Western blot analysis. Antibodies used were as follows: HIF-1α antibody was from Cell Signaling Technology (rabbit, #14179, 1:1000 dilution), and HIF-2α antibody (rabbit, #PA1129-2, 1:1000 dilution) was from Boster Bio. The antibody against α2-NKA (rabbit, #ANP-002, 1:1000 dilution) was from Alomone Labs and beta-actin, used to normalize the band densities as a housekeeping gene (mouse, #A2228, 1:10000 dilution), was from Sigma. Anti-mouse (GE Healthcare/Amersham, Amersham, UK, #NA931, 1:10,000) or anti-rabbit (Sigma, #401315, 1:2000) secondary antibodies conjugated with horseradish peroxidase and enhanced chemiluminescence (Amersham, #GE.RPN2232) were used for detection. Band densities were measured using the Image J 1.42q software (NIH, Bethesda, MD, USA).

### 2.7. Statistical Analysis

Results are shown as mean ± SD as indicated. Statistical analysis was performed by analysis of variance (ANOVA) for repeated measures and pair-wise multiple comparisons (LSD) or *t*-tests, as indicated, using the SigmaPlot 10.0 (Systat Inc., Erkrath, Germany) software package. The level of statistical significance was set as *p* < 0.05.

## 3. Results

### 3.1. Silencing Efficiency of HIFs on the mRNA Expression of HIF-1α and HIF-2α

**Figure 1:**
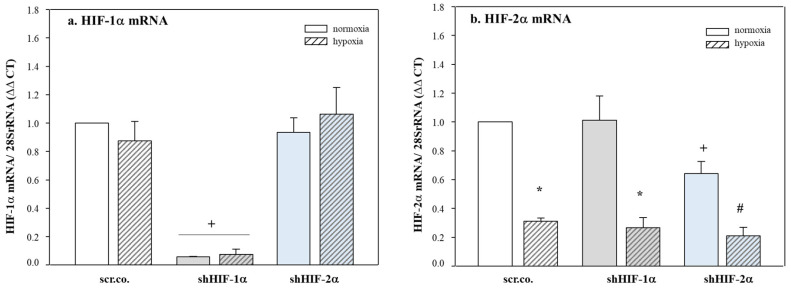
**Effect of hypoxia and HIF silencing on the expression of HIF mRNA.** H9c2 cells were infected with 100 MOI shHIF-1α (**a**) and shHIF-2α (**b**) containing adenoviral vectors and kept in normoxia (19% O_2_) or hypoxia (1% O_2_) for 24 h. mRNA expression was normalized to 28SrRNA, and the results are given as the normalized values of scr.co. Mean values ± SD of four independent experiments normalized to normoxic scr.co are shown. The level of significance was set as *p* < 0.05: + effect of silencing compared to respective controls *p* < 0.001, * effect of hypoxia compared to normoxic respective controls *p* < 0.05, # effect of HIF-2α silencing compared to hypoxic scr.co. scr.co: scrambled control; shHIF-1α: HIF-1α-silenced cells; shHIF-2α: HIF-2α-silenced cells.

Silencing HIF-1α decreased the HIF-1α mRNA expression in normoxic and hypoxic cells by 95% (*p* < 0.001) compared to the respective controls (Figure 1a). Hypoxia decreased the mRNA expression of HIF-2α by 80% compared to the normoxic control cells (*p* = 0.008). Silencing HIF-2α decreased HIF-2α mRNA by ~40% in normoxic cells (*p* = 0.001) and by ~30% in hypoxic cells (*p* = 0.007, Figure 1b). A higher amount of adenoviral vector treatment did not improve the efficiency of HIF-2α silencing.

### 3.2. Silencing Efficiency of HIFs on the Protein Expression of HIF-1α and HIF-2α

**Figure 2:**
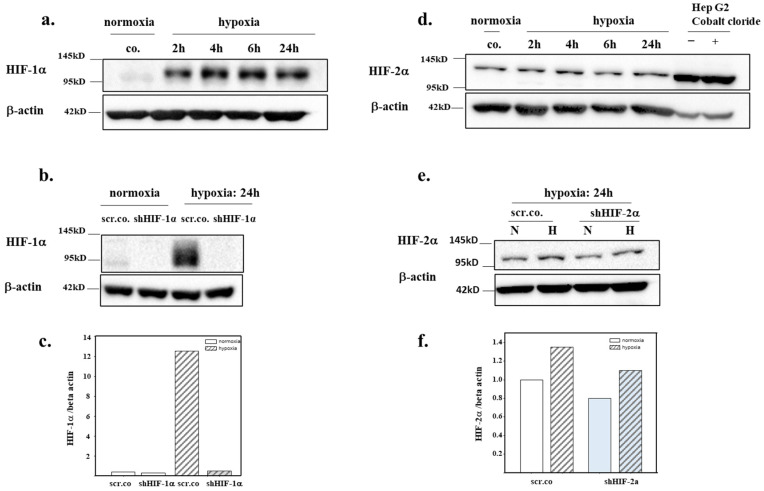
**Efficiency of HIF silencing on the nuclear accumulation of HIF-1α and HIF-2α.** H9c2 were kept in normoxia (19% O_2_) and hypoxia (1% O_2_) for 2 h, 4 h, 6 h, 24 h (**a**,**c**). A total of 10-25 µg of nuclear extract protein was used for the analysis of HIF-α by Western blotting. H9c2 cells were silenced with 100 MOI shHIF-1α (**b**,**c**) and shHIF-2α (**e**,**f**) containing adenoviral vectors and kept in normoxia (19% O_2_) or hypoxia (1% O_2_) for 24 h. Representative immunoblots showing the silencing efficiency of HIF-1α (**b**) and of HIF-2α (**d**). scr.co: scrambled control; shHIF-1α: HIF-1α-silenced cells; shHIF-2α: HIF-2α-silenced cells.

To test the time dependency and maximum expression of HIF-α at 1% O_2_, we kept the cells in hypoxia for 2 h, 4 h, 6 h, and 24 h. HIF-1α protein accumulation in the nuclear extracts was evident at 2 h of hypoxia and persisted for 24 h (Figure 2a). Silencing HIF-1α totally inhibited HIF-1α protein expression in hypoxia-exposed cells within 24 h (Figure 2b,c). Figure 2d,e shows that HIF-2α is constitutively expressed in normoxic cells. Hypoxia for 24 h increased HIF-2α accumulation by ~30% and was fully inhibited by silencing compared to the hypoxic controls (Figure 2e,f).

### 3.3. Effect of Hypoxia and HIF Silencing on the mRNA Expression of α2-NKA

**Figure 3:**
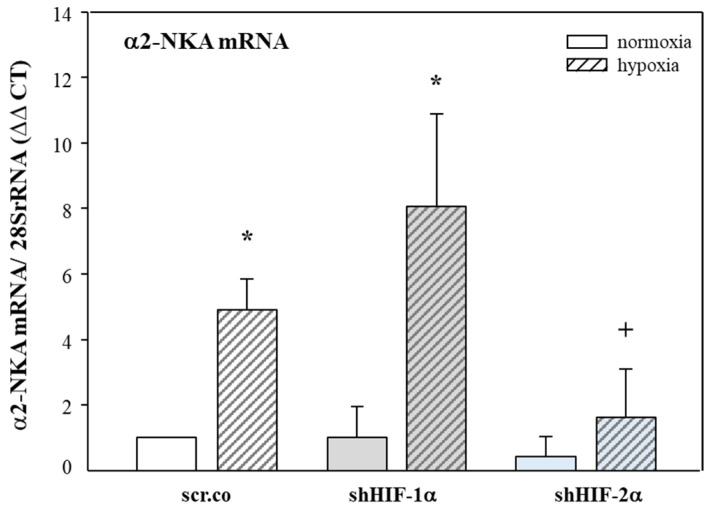
**Effects of hypoxia and HIF silencing on α2-NKA mRNA expression.** H9c2 cells were infected with 100 MOI shHIF-1α and shHIF-2α containing adenoviral vectors and kept in normoxia (19% O_2_) or hypoxia (1% O_2_) for 24 h. mRNA expression was normalized to 28SrRNA, and the results are given as the normalized values of scr.co. Mean values ± SD of four independent experiments as shown. The level of significance was set to *p* < 0.05: * effect of hypoxia compared to normoxia, + effect of HIF-2α silencing in hypoxic cells compared to hypoxic scr.co. scr.co: scrambled control; shHIF-1α: HIF-1α-silenced cells; shHIF-2α: HIF-2α-silenced cells.

Figure 3 shows that hypoxia increased the mRNA expression of α2-NKA compared to normoxic cells by 5-fold (*p* = 0.007). Silencing HIF-2α decreased the α2-NKA mRNA expression by ~70% (*p* = 0.038) compared to hypoxia, but the increase by hypoxia seen in the controls and HIF-1α-silenced cells prevailed. There was no effect of HIF-1α silencing on α2-NKA mRNA expression. These results indicate that mRNA expression of α2-NKA in H9c2 cells is controlled by HIF-2α, both in normoxia and hypoxia.

### 3.4. Effect of Hypoxia and HIF Silencing on α2-NKA Protein Expression and Membrane Insertion

**Figure 4:**
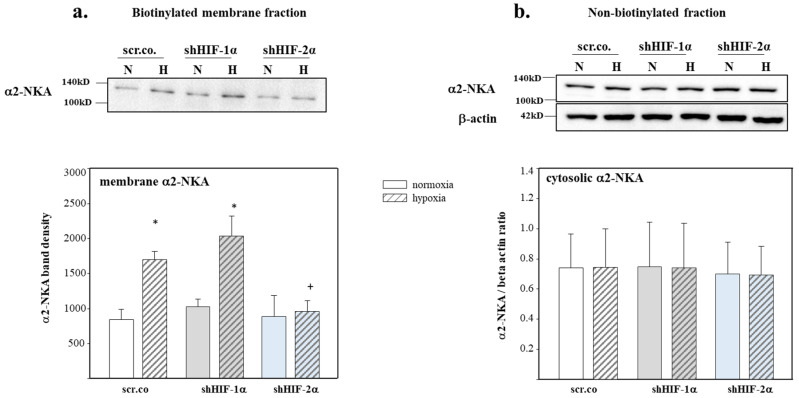
Effect of hypoxia and HIF silencing on the abundance of α2-NKA in the membranes and cytoplasmic fractions of H9c2 cells. Plasma membrane and intracellular α2-NKA protein expression of H9c2 cells silenced with shHIFs and kept in normoxia or hypoxia. The biotinylated fraction is considered the membrane fraction, and the non-biotinylated is considered the intracellular fraction. A representative blot showing α2-NKA (**a**) surface expression (biotinylated) and (**b**) intracellular expression (non-biotinylated). Beta-actin was not detected in the surface (biotinylated) membrane fraction but only in the intracellular pool. Mean values ± SD of four independent experiments as shown. The level of significance was set at *p* < 0.05: * effect of hypoxia compared to normoxia, + effect of HIF-2α silencing in hypoxic cells compared to hypoxic scr.co. scr.co: scrambled control; shHIF-1α: HIF-1α-silenced cells; shHIF-2α: HIF-2α-silenced cells.

Next, we tested to what extent intracellular trafficking of α2-NKA was affected by hypoxia and HIFs. Figure 4a shows a 2-fold increase in the plasma membrane abundance of α2-NKA in hypoxic cells compared to the normoxic controls (*p* = 0.005). Silencing HIF-2α fully prevented this effect (*p* = 0.006), whereas there was no effect of silencing HIF-1α (*p* = 0.269). Figure 4b shows α2-NKA protein in the non-biotinylated fractions, which indicates α2-NKA expression in the cytosol is not affected by hypoxia or HIF silencing. These data indicate that the increased membrane protein turnover of α2-NKA in hypoxic H9c2 cardiomyocytes is controlled by HIF-2α.

## 4. Discussion

The major finding of this study is that 24 h hypoxia exposure of H9c2 cardiomyocytes increased the mRNA expression of α2-NKA and its membrane abundance. Furthermore, we showed here, for the first time, that HIF-2α controls both the mRNA expression and membrane abundance of α2-NKA because silencing HIF-2α prevented this increase.

Owing to the essential roles of NKA in the cardiovascular system, its function and regulation has been extensively investigated on patient samples with different disease backgrounds and various animal models of ischemic heart disease that focus on the NKA activity and subunit expression. So far, we know that NKA activity decreases in ischemic/hypoxic heart tissue, but the underlying mechanisms are not clear.

The decreased NKA activity has been attributed to decreased expression of the subunit proteins. However, inconsistent findings from expression studies cannot substantially clarify the decreased NKA activity [2]. For instance, in a rabbit heart failure model, Bossuyt et al. reported increased intracellular Na^+^ accumulation, decreased NKA activity along with decreased protein expression of α1- and α2-NKA in ventricular homogenates and isolated myocytes. This study also found increased expression of α3-NKA in whole heart homogenates but decreased expression in myocytes isolated from the same animals [18]. A similar result was found in rats with cardiac contractile dysfunction induced by chronic renal failure after partial nephrectomy [36]. In contrast to these reports, Allen et al. showed no change in the α1-, α2-, and α3-NKA mRNA or protein expression in ventricular tissues from normal or end-stage heart failure patients [22], whereas Jager et al. reported a 5-fold increase in α2-NKA mRNA expression in the right atrium of patients with hypertension. However, this study did not report protein expression [21]. Another study by Magyar et al. in a renovascular hypertension model showed unaltered α1-NKA but decreased α2-NKA mRNA and protein levels [23].

One likely reason for these discrepant findings in human studies might be due to patient backgrounds, stage of the diseases, drugs used, part of the specimens taken, and a different degree of hypoxic stress existing that might contribute bias to findings. In animal models, different animal species, models, and tissue type used for the measurements might contribute to inconsistencies as well.

Because of the difficulty to achieve conditions of comparable degrees of hypoxia and model-related cardiac injury, an invitro model of hypoxic myocardium might help provide a clearer picture on the changes and mechanisms. In our study we used H9c2 rat ventricular cardiomyocytes, one of the most used cells in experiments concerning cardiomyocyte cellular functions.

Concerning the inhomogeneous results on NKA expression and activity, only a few studies have dealt with the potential mechanisms. One unifying mechanism might be hypoxia, which is common in all models and hypoxia-induced cell signalling in cardiovascular diseases (reviewed recently in [2,37,38]). However, information on hypoxia and HIFs in regulating ion channels and transporters in ischemic/hypoxic heart tissue is scarce. Here, we show for the first time that mRNA and membrane expression of α2-NKA is increased in hypoxic cardiomyocytes, which suggest that hypoxia increases the transcription and translation of α2-NKA, leading to increased membrane translocation and expression. Silencing HIF-2α prevented all these effects. The mechanism behind HIF-2α-dependent regulation of α2-NKA is not yet known. So far, there is no information on whether rat α2-NKA has a Hypoxia-Response Element (HRE) binding site in the promoter region. It also remains to be identified whether the effects observed in our study are direct or indirect effects of HIF-2α. Future studies are required to unravel the HIF-2α dependency of α2-NKA in hypoxic myocardium.

Studies on hypoxia-HIF-dependent modulation of the activity and expression of signalling molecules regulating myocardial ion homeostasis and contraction are scarce. Ronkainen et al., using embryonic mouse cardiomyocytes, showed that hypoxia decreased SERCA2a expression and activity, impaired cardiac contractility, and caused arrhythmia. Overexpression of HIF-1α in normoxic cells and chemical activation of HIF-1α mimicked the findings induced by hypoxia. This study identified HRE binding sites in the SERCA2 promoter region [39] that might explain the HIF-1α dependency. In a myocardial infarction model, CaMK2γ expression decreased due to elevated HIF-1α levels, pointing to its important role in the transcriptional response to hypoxia [40]. In a recent report, we showed in in-vitro and in-vivo models of pulmonary oedema that hypoxic impairment of lung fluid clearance and decreased plasma membrane expression of epithelial Na channels were depended on HIF-2α activity [34]. In the current study, we did not find any effect of HIF-1α on the mRNA and protein expression of α2-NKA. However, it needs to be identified whether the expression of other NKA subunits changes in hypoxia and whether HIFs are involved in regulating their expression.

In addition, the results obtained from this study need to be tested on the cellular level in primary human cardiomyocytes and in in-vivo models of ischemic heart disease because the H9c2 cell line used in this study does not fully match mature cardiomyocytes.

Taken together, our results show for the first time that α2-NKA expression and protein abundance in the plasma membrane are increased in hypoxic cardiomyocytes by HIF-2α-dependent mechanisms. It needs to be clarified whether hypoxia mediated increased expression of α2-NKA is a consequence of the adaption to hypoxia or a compensatory mechanism for decreased NKA activity even at the high cost of ATP consumption. The mechanism behind this, molecular basis of the behaviour of α2-NKA in cardiac disease models, and any effect on the function of other Na^+^ and Ca^2+^ transporting systems need further investigation. Given that NKA activity decreases in heart failure and endogenous cardiotonic steroid levels increase in ischemic heart disease [41,42], exogenous use of cardiotonic steroids might further aggravate the dysregulated myocardial Ca^2+^ and Na^+^ ion homeostasis due to their high binding affinity to α2-NKA. Therefore, mechanisms of both effects and side effects of cardiotonic steroids in ischemic heart disease need to be identified for their precise use in clinics.

## Figures and Tables

**Table 1:** List of the primers.

Name	Catalogue Number
rat 28SrRNA	Rn_Rnr1_1_SG QuantiTect Primer Assay QT00199374
rat HIF-1α	Rn_Hif1a_1_SG QuantiTect Primer Assay QT00182532
rat HIF-2α	Rn_Epas1_1_SG QuantiTect Primer Assay QT00192059
rat α2-NKA	Rn_Atp1a2_1_SG QuantiTect Primer Assay QT00175924

## Data Availability

Not applicable.
